# Characteristics and outcomes of patients undergoing transcatheter mitral valve replacement with the Tendyne system

**DOI:** 10.1007/s00392-023-02155-x

**Published:** 2023-01-16

**Authors:** Nihal Wilde, Tetsu Tanaka, Vivian Vij, Atsushi Sugiura, Mitsumasa Sudo, Eva Eicheler, Miriam Silaschi, Johanna Vogelhuber, Farhad Bakhtiary, Georg Nickenig, Marcel Weber, Sebastian Zimmer

**Affiliations:** 1 Heart Center Bonn, Department of Internal Medicine II, University Hospital Bonn, Venusberg-Campus 1, 53127 Bonn, Germany; 2 Heart Center Bonn, Department of Cardiac Surgery, University Hospital Bonn, Bonn, Germany

**Keywords:** Mitral valve disease, Mitral regurgitation, Transcatheter mitral valve replacement, Tendyne system, Transcatheter edge-to-edge repair

## Abstract

**Background:**

Transcatheter mitral valve replacement (TMVR) has emerged as alternative to transcatheter edge-to-edge repair (TEER) for treatment of mitral regurgitation (MR); however, the role of TMVR with the Tendyne system among established treatments of MR is not well defined. We assessed characteristics and outcomes of patients treated with the Tendyne system in the current clinical practice.

**Methods:**

We reviewed patients who underwent cardiac computed tomography and were judged eligible for the Tendyne system.

**Results:**

A total of 63 patients were eligible for TMVR with the Tendyne system. Of these, 17 patients underwent TMVR, and 46 were treated by TEER. Patients treated with the Tendyne system were more likely to have a high transmitral pressure gradient and unsuitable mitral valve morphology for TEER than those treated with TEER. TMVR with the Tendyne system reduced the severity of MR to less than 1 + in 94.1% of the patients at discharge and achieved a greater reduction in left ventricular (LV) end-diastolic volume at the 30-day follow-up compared with TEER. In contrast, patients treated with the Tendyne system had a higher 30-day mortality than those treated with TEER, while the mortality between 30 days and one year was comparable between Tendyne and TEER.

**Conclusions:**

Among patients eligible for the Tendyne system, approximately a quarter of the patients underwent TMVR with the Tendyne system, which led substantial reduction of MR and LV reverse remodeling than TEER. In contrast, the 30-day mortality rate was higher after TMVR with the Tendyne compared to TEER.

**Supplementary Information:**

The online version contains supplementary material available at 10.1007/s00392-023-02155-x.

## Introduction

Mitral regurgitation (MR) is the most common valvular heart disease and is associated with an increased risk of mortality and poor quality-of-life, irrespective of the underlying anatomy or etiology of the MR [1]. Transcatheter edge-to-edge repair (TEER) is an emerging treatment option for patients with symptomatic MR that have a high risk for surgery [2–4]. Reduction of MR by use of TEER is associated with improved clinical outcome [5]. However, the effectiveness of TEER is predicated on the underlying anatomy of the mitral valve [6].

Currently, transcatheter mitral valve replacement (TMVR) is being developed as an alternative treatment [7, 8]. The Tendyne Mitral Valve System (Abbott Vascular, Roseville, MN, USA) is the first commercially available TMVR device approved in Europe 2020 [9]. Previous studies have shown that TMVR with the Tendyne system is safe and feasible, and that it can achieve complete elimination of MR [10, 11]. TMVR with the Tendyne system may have a potential to address the unmet clinical needs for patients with suboptimal mitral valve anatomy for TEER. Furthermore, given its potential for complete MR abolishment, the Tendyne system might lead to greater benefits for both clinical prognosis and left-ventricular (LV) reverse remodeling, as compared to TEER.

The role of the Tendyne system among established treatments for MR has not yet been clearly defined. There is an increasing demand for a more refined device selection for the transcatheter mitral valve treatments. Insight into patient selection and procedural results of the Tendyne system in the current clinical practice, as well as their comparison with TEER, would help with further refinement of the device selection for transcatheter treatments. Therefore, we conducted this observational study to assess the characteristics and outcomes of patients undergoing TMVR with the Tendyne system in comparison with those undergoing TEER.

## Methods

### Study population

**Fig. 1:**
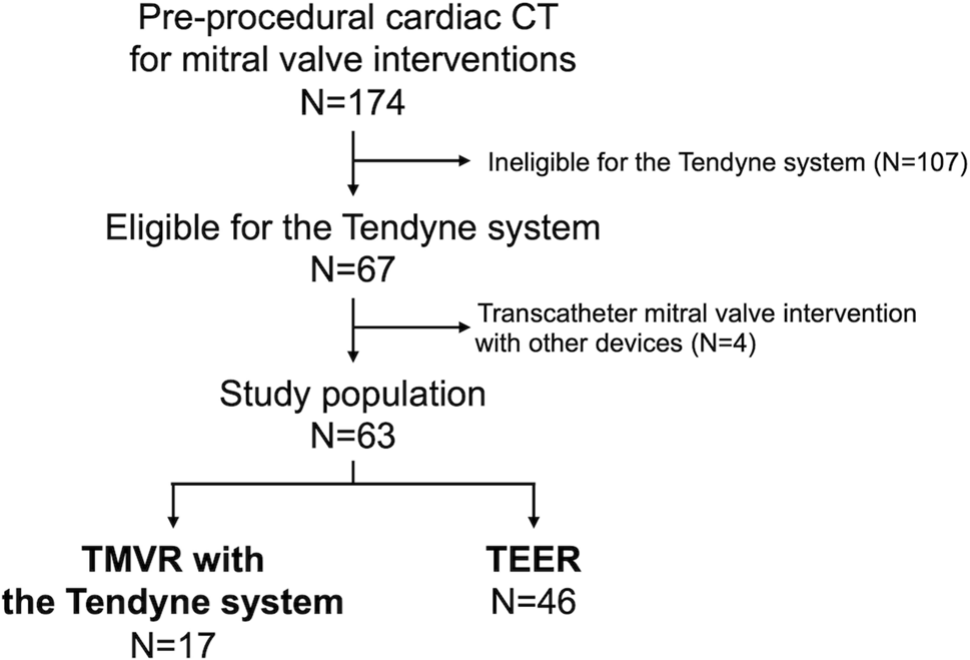
Study flowchart. *CT*  computed tomography, *TMVR*  transcatheter mitral valve replacement, *TEER*  transcatheter edge-to-edge repair

This is a retrospective study based on the data from the Bonn registry, which is a prospective, consecutive collection of patient data from the Heart Center Bonn. We identified patients with symptomatic MR who underwent cardiac computed tomography (CT) for the pre-procedural assessments of transcatheter mitral valve interventions from April 2019 to October 2021. All included patients were considered as inoperable or at high-risk for surgery by the interdisciplinary heart team. After a standardized diagnostic workup, including transthoracic (TTE) and transesophageal echocardiography (TEE) and left-heart catheterization, the patient’s anatomical suitability for the Tendyne system was assessed using the CT images. Grading of mitral annular calcification in mild, moderate and severe was performed according to the CT-based scoring system as previously reported [12]. The current analysis included patients who were considered to be eligible for the Tendyne system and underwent the transcatheter mitral valve interventions with the Tendyne system or TEER devices (Fig. 1).

The indication for a mitral valve intervention was evaluated based on the current guidelines [13], and the decision to perform transcatheter mitral valve interventions was made by the interdisciplinary heart team. Recommended device of mitral valve intervention was decided in each individual by the heart team, considering the morphology of mitral valve, cardiac function, clinical characteristics, and patients’ background and preference. The registry was approved by the local ethics committee. This study was conducted in accordance with the Declaration of Helsinki and its amendment, and all patients provided written informed consent.

### Procedure

The TMVR procedure with the Tendyne Mitral Valve System (Abbott Vascular, Roseville, MN, USA) was performed under general anesthesia with 3D-TEE and fluoroscopic guidance, as previously reported [9]. In brief, using a left lateral mini-thoracotomy, the prosthesis is placed in the native mitral valve annulus and retained by a tether connected to an epicardial pad, which is fixed on the apex according to the counter-pull principle. The prosthesis was repositioned and retrieved if the positioning was found to be suboptimal, such as in the case of left-ventricular outflow tract obstruction. Sizes and profiles of the device were chosen according to the individual patient’s anatomy, as assessed by TTE, TEE, and CCT before the procedure.

The TEER procedure was performed with either the MitraClip system (Abbott Structural Heart, Santa Clara, CA, USA) or PASCAL system (Edwards Lifesciences, Irvine, CA, USA). The details of each device system and procedure have previously been well described [14, 15]. Technical success was defined using Mitral Valve Academic Research Consortium (MVARC) criteria [16].

### Echocardiographic assessments

Comprehensive TTE and TEE were performed at baseline and discharge, according to the current guidelines. Based on the quantitative assessments in the current guideline [17], the severity of MR was graded as follows: grade 0, none; 1 + , mild; 2 + , moderate; 3 + , moderate-to-severe; 4 + , severe. LV volumes and ejection fractions were assessed at the apical biplane views, including two- and four-chamber views. Also, the suitability of mitral valve morphology for TEER was evaluated based on the German criteria [18]. Post-procedural echocardiographic assessments were collected at 30 days after the procedure. Changes in LV end-diastolic and end-systolic volumes from baseline to 30 days were evaluated as follows: (LV volume at baseline – LV volume at the 30-day follow-up) / LV volume at baseline. All measurements were reviewed by two independent cardiologists dedicated to echocardiographic evaluation.

### Follow-up

All-cause mortality within one year after the procedure was assessed. The severity of MR and changes in LV end-diastolic and end-systolic volumes from baseline to 30 days were also evaluated. Adverse events were adjudicated by the local heart team according to the criteria of the Mitral Valve Academic Research Consortium [16]. All patients were followed-up through interviews at scheduled hospital visits, telephone, or documentation from the referring general practitioners.

### Statistics

Continuous variables are presented as the mean ± standard deviation or the median with an interquartile range (IQR) and compared using the Mann–Whitney test. Proportions of categorical data are presented as numbers with percentages and compared using Fisher’s exact test. A Wilcoxon signed-rank test was used to compare variables between two time points. We carried out inverse probability of treatment weighting (IPTW)-adjusted analyses to balance the clinical characteristics between the Tendyne and TEER devices [19]. Propensity score was calculated for each patient using multivariate logistic regression that estimates the propensity toward belonging to a specific treatment group (Tendyne versus TEER devices). The following covariates were included in the multivariable model: age, male sex, body mass index (BMI), end-stage renal dysfunction requiring hemodialysis, etiology of MR, mean transmitral pressure gradient, LV ejection fraction (LVEF), and LV end-diastolic volume. Each patient was weighted by the inverse probability of being in the observed group. After IPTW, survival curves within one year after the procedure are depicted using the Kaplan–Meier method and compared using a log-rank test. By setting a landmark at 30 days, we compared the mortality from 30 days to one year between the groups using Kaplan–Meier curves. Details of the IPTW analysis are shown in the Supplement material. Statistical significance was set as a two-sided *p*-value < 0.05. All analyses were conducted using Stata/SE 15.1 (StataCorp, College Station, TX, USA).

## Results

### Study population

Among the 174 patients who underwent CT to assess the anatomical eligibility for TMVR with the Tendyne system between April 2019 and October 2021, 67 patients were considered to have suitable anatomy of the mitral valve and LV for the Tendyne system (Fig. 1). Of these, 17 patients underwent TMVR with the Tendyne system, and 46 underwent TEER with the MitraClip or PASCAL systems (Fig. 1). Four patients who underwent mitral valve interventions with other devices were excluded from this analysis.

Overall, the mean age was 76.7 ± 7.9 years, 46.0% of patients were male, and a secondary etiology of MR was observed in 63.5% of the patients (Table 1). The median EuroSCORE II was 7.5 ± 5.8%. Based on the German criteria, 20 patients (31.7%) were considered as having optimal mitral valve morphology for TEER, while 10 patients (15.9%) had unsuitable valve morphology.

**Table 1:** Baseline patient characteristics

	All	Tendyne	TEER	*p* value
	*N* = 63	*N* = 17	*N* = 46	
Age, years	76.7 ± 7.9	72.9 ± 9.4	78.1 ± 6.9	0.021
Male, *n* (%)	29 (46.0)	8 (47.1)	21 (45.7)	> 0.99
BMI, kg/m^2^	27.4 ± 6.4	30.7 ± 7.4	26.1 ± 5.6	0.011
CAD, *n* (%)	36 (57.1)	9 (52.9)	27 (58.7)	0.78
Prior MI, *n* (%)	8 (12.7)	2 (11.8)	6 (13.0)	> 0.99
Prior cardiac surgery, *n* (%)	18 (28.6)	5 (29.4)	13 (28.3)	> 0.99
COPD, *n* (%)	20 (31.7)	5 (29.4)	15 (32.6)	> 0.99
NYHA IV, *n* (%)	11 (17.5)	2 (11.8)	9 (19.6)	0.71
Atrial fibrillation, *n* (%)	49 (77.8)	13 (76.5)	36 (78.3)	> 0.99
CIED, *n* (%)	15 (23.8)	4 (23.5)	11 (23.9)	> 0.99
eGFR, ml/min/m^2^	53.8 [39.8, 70.4]	53.0 [34.8, 66.8]	53.9 [40.8, 72.6]	0.39
Hemodialysis, *n* (%)	4 (6.4)	4 (23.5)	0 (0.0)	0.004
NT-proBNP, pg/ml	1866 [1214, 4147]	1582 [1285, 5035]	2528 [1175, 3953]	0.56
EuroSCORE II, %	7.5 ± 5.8	7.6 ± 6.1	7.5 ± 5.7	0.96
Beta blockers, *n* (%)	53 (84.1)	12 (70.6)	41 (89.1)	0.12
RAS inhibitors, *n* (%)	51 (81.0)	14 (82.4)	37 (80.4)	> 0.99
MRAs, *n* (%)	22 (34.9)	4 (23.5)	18 (39.1)	0.22
Diuretics, *n* (%)	55 (87.3)	13 (76.5)	42 (91.3)	0.17
*Echocardiographic findings*				
LVEF, %	55.6 [43.1, 59.0]	55.5 [52.2, 58.0]	55.7 [43.1, 59.5]	0.67
LV end-diastolic volume, ml	115.6 [93.9, 147.5]	124.7 [107.5, 152.2]	114.3 [91.0, 146.5]	0.29
LV end-systolic volume, ml	53.7 [38.8, 69.4]	57.3 [43.2, 69.4]	60.4 [37.0, 68.1]	0.69
LV length, mm	78 [72, 84]	81 [77, 84]	77 [72, 83]	0.22
LA volume, ml	91.8 [66.9, 120.0]	100.0 [77.9, 120.0]	90.0 [61.2, 117.8]	0.25
Etiology of MR: FMR, n (%)	40 (63.5)	8 (47.1)	32 (69.6)	0.14
*MR severity, n* (%)				> 0.99
3 +	24 (38.1)	6 (35.3)	18 (39.1)	
4 +	39 (61.9)	11 (64.7)	28 (60.9)	
EROA, mm^2^	35 [29, 42]	38 [31, 42]	34 [29, 41]	0.71
Regurgitant volume, ml	56 [45, 71]	56 [41, 76]	55 [45, 71]	0.57
Mean transmitral pressure gradient, mmHg	2.0 [1.2, 3.0]	2.2 [1.8, 4.0]	1.8 [1.1, 2.7]	0.039
Posterior leaflet length < 10 mm, *n* (%)	15 (23.8)	6 (35.3)	9 (19.6)	0.20
Coaptation depth ≥ 11 mm, *n* (%)	6 (9.5)	1 (5.9)	5 (10.9)	> 0.99
*Mitral valve morphology for TEER, n* (%)				0.16
Optimal	20 (31.7)	4 (23.5)	16 (34.8)	
Conditionally suitable	33 (52.4)	8 (47.1)	25 (54.3)	
Unsuitable	10 (15.9)	5 (29.4)	5 (10.9)	
SPAP, mmHg	41 [30, 46]	40 [38, 45]	41 [28, 47]	0.43
TAPSE, mm	20 [15, 23]	20 [15, 27]	20 [15, 22]	0.66
TR: severe or more, *n* (%)	22 (34.9)	5 (29.4)	17 (37.0)	0.77
*CT findings*				
MV annular area, cm^2^	12.5 ± 2.0	12.2 ± 2.3	12.7 ± 2.0	0.42
MV annular perimeter, mm	130 ± 10	129 ± 12	131 ± 10	0.45
MV annular antero-posterior diameter, mm	35.7 ± 3.6	34.8 ± 4.4	36.0 ± 3.3	0.25
MV annular intercommissural diameter, mm	42.1 ± 4.2	41.7 ± 3.0	42.2 ± 4.5	0.62
*Mitral annular calcification, n* (%)				0.62
None	36 (57.1)	9 (52.9)	27 (58.7)	
Mild	17 (27.0)	6 (35.3)	11 (23.9)	
Moderate	7 (11.1)	2 (11.8)	5 (10.9)	
Severe	3 (4.8)	0 (0.0)	3 (6.5)	

Values are either *n* (%), mean ± SD, or median [interquartile range]

*TEER*  transcatheter edge-to-edge repair, *BMI*  body mass index, *CAD*  coronary artery disease, *MI*  myocardial infarction, *COPD*  chronic obstructive pulmonary disease, *NYHA*  New York Heart Association, *CIED*  cardiac implantable electronic device, *eGFR*  estimated glomerular filtration rate, *NT-proBNP*  N-terminal pro-B-type natriuretic peptide, *EuroSCORE*  European System for Cardiac Operative Risk Evaluation, *RAS*  renin-angiotensin system, *MRA*  mineralocorticoid receptor antagonist, *LVEF*  left-ventricular ejection fraction, *LA*  left-atrium, *FMR*  functional mitral regurgitation, *SPAP*  systolic pulmonary artery pressure, *TAPSE*  tricuspid annular plane systolic excursion, *TR*  tricuspid regurgitation, *CT*  computed tomography, *MV*  mitral valve

### Characteristics of patients treated with the Tendyne system

In 17 patients treated with the Tendyne system, the mean age was 72.9 ± 9.4 years, 47.1% of patients were male, and a secondary etiology of MR was observed in 47.1% of the patients (Table 1). Based on the German criteria, 29.4% of the patients had unsuitable mitral valve morphology for TEER. Eight of the 17 patients in the Tendyne group had a prior history of an unsuccessful TEER procedure.

Patients treated with the Tendyne system were younger and had a higher body mass index and a higher frequency of end-stage renal dysfunction requiring hemodialysis, compared with patients treated with TEER. Furthermore, the Tendyne group was likely to have a higher mean transmitral pressure gradient and a primary etiology of MR than the TEER group. Based on the German criteria, patients treated with the Tendyne appeared to have unsuitable valve morphology for TEER compared to those treated with TEER (29.4% vs. 10.9%; *p* = 0.10). In contrast, LV ejection fraction, LV chamber size, and severity of MR were comparable between the Tendyne and TEER groups. CT assessment showed that the anatomy of mitral annulus and the severity of mitral annular calcification were comparable between the two groups.

### Procedural outcomes and in-hospital clinical events

Periprocedural findings are summarized in Table 2. Technical success was achieved in 94.1% of patients treated with the Tendyne system. Of the 17 patients treated with the Tendyne system, one experienced aborted valve implantation because of acutely worsening aortic regurgitation after the valve implantation which was not solved by repositioning the valve or by use of a lower profile valve. No left-ventricular outflow tract obstruction occurred.

**Table 2:** Procedural findings and in-hospital adverse events

	All	Tendyne	TEER	*p* value
	*N* = 63	*N* = 17	*N* = 46	
Technical success, *n* (%)	59 (93.7)	16 (94.1)	43 (93.5)	> 0.99
Number of implanted clips	1.3 ± 0.6	–	1.3 ± 0.6	–
*MR severity after procedure, n* (%)				< 0.001
0	16 (25.4)	16 (94.1)	1 (2.2)	
1 +	36 (57.1)	0 (0.0)	35 (76.0)	
2 +	6 (9.5)	1 (5.9)	5 (10.9)	
3 +	4 (6.4)	0 (0.0)	4 (8.7)	
4 +	1 (1.6)	0 (0.0)	1 (2.2)	
Mean transmitral pressure gradient, mmHg	3.7 [2.8, 4.7]	4.2 [2.9, 4.7]	3.7 [2.7, 4.6]	0.70
*In-hospital events*				
In-hospital mortality, *n* (%)	3 (4.8)	3 (17.7)	0 (0.0)	0.018
Stroke, *n* (%)	2 (3.2)	1 (5.9)	1 (2.2)	0.47
Major or life-threating bleeding, *n* (%)	3 (4.8)	1 (5.9)	2 (4.4)	> 0.99
Sepsis, *n* (%)	3 (4.8)	3 (17.7)	0 (0.0)	0.017

*TEER*  transcatheter edge-to-edge repair, *MR*  mitral regurgitation

Values are either *n* (%), mean ± SD, or median [interquartile range]

During the hospital stay, one of 17 patients treated with the Tendyne system experienced a disabling stroke, one experienced acute kidney failure that required dialysis, and three experienced sepsis (Table 2). Major bleeding events occurred in two patients, including a case requiring an open-heart surgery due to pericardial bleeding. Three out of the 17 patients experienced in-hospital mortality, including two due to respiratory infection and subsequent sepsis and one due to multi-organ dysfunction after an open-heart surgery.

### Echocardiographic assessments after the procedure

Post-procedural echocardiographic assessments were available in all patients (Table 2), and 59 of the 63 study participants underwent echocardiographic assessments at 30 days after the procedure (Table 3).

**Table 3:** Echocardiographic assessment at the 30-day follow-up

	Tendyne	TEER	*p* value
	*N* = 14	*N* = 45	
LVEF, %	54.3 [45.9, 56.5]	55.6 [50.0, 59.7]	0.22
LV end-diastolic volume, ml	98.8 [70.9, 117.3]	106.0 [85.8, 141.6]	0.16
Percent change from baseline, %	27.3 [ – 0.4, 41,9]	5.0 [ – 30.9, 17.9]	0.033
LV end-systolic volume, ml	41.0 [32.8, 57.1]	50.1 [35.6, 70.2]	0.40
Percent change from baseline, %	5.1 [ – 2.7, 43.2]	7.0 [ – 30.1, 22.9]	0.31
LV length, mm	71 [67, 80]	74 [68, 78]	0.58
LA volume, ml	113.8 [83.9, 128.1]	83.3 [65.4, 107.2]	0.12

Values show the median [interquartile range]

*TEER*  transcatheter edge-to-edge repair, *LVEF*  left-ventricular ejection fraction, *LA*  left atrium

**Fig. 2:**
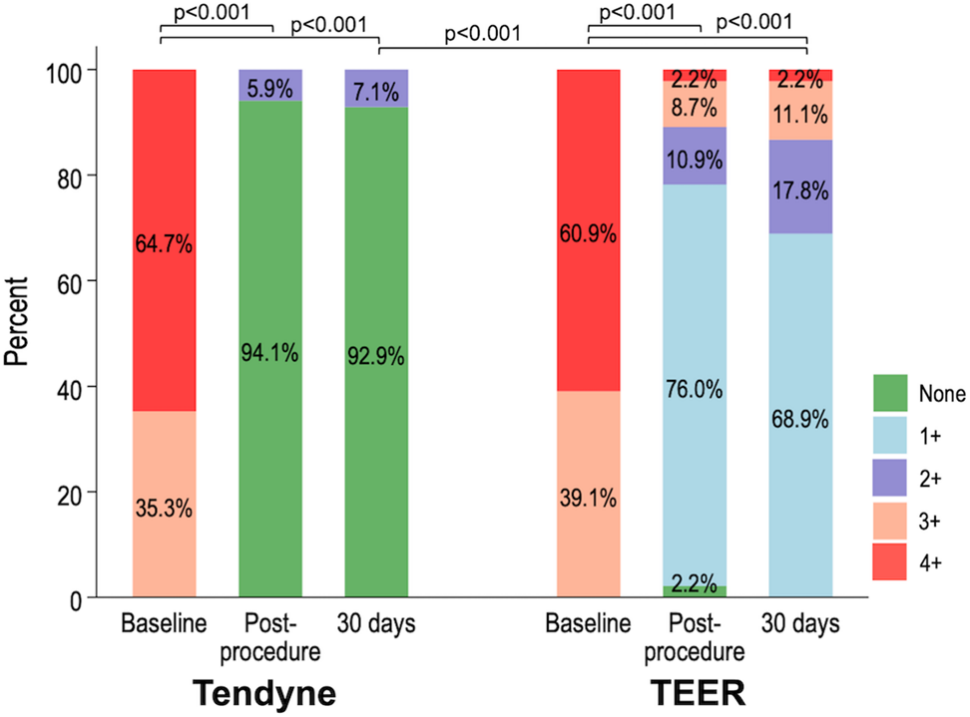
Changes in the severity of mitral regurgitation after the procedure. The severity of mitral regurgitation at baseline, post-procedure, and 30 days after the procedure in the Tendyne and transcatheter edge-to-edge repair (TEER) groups

Post-procedural echocardiographic assessments showed that the severity of MR reduced to less than 1 + in 94.1% of patients treated with the Tendyne system (Fig. 2). The reduction of MR in the Tendyne group was consistent at the 30-day follow-up, which was greater than in the TEER group.

**Fig. 3:**
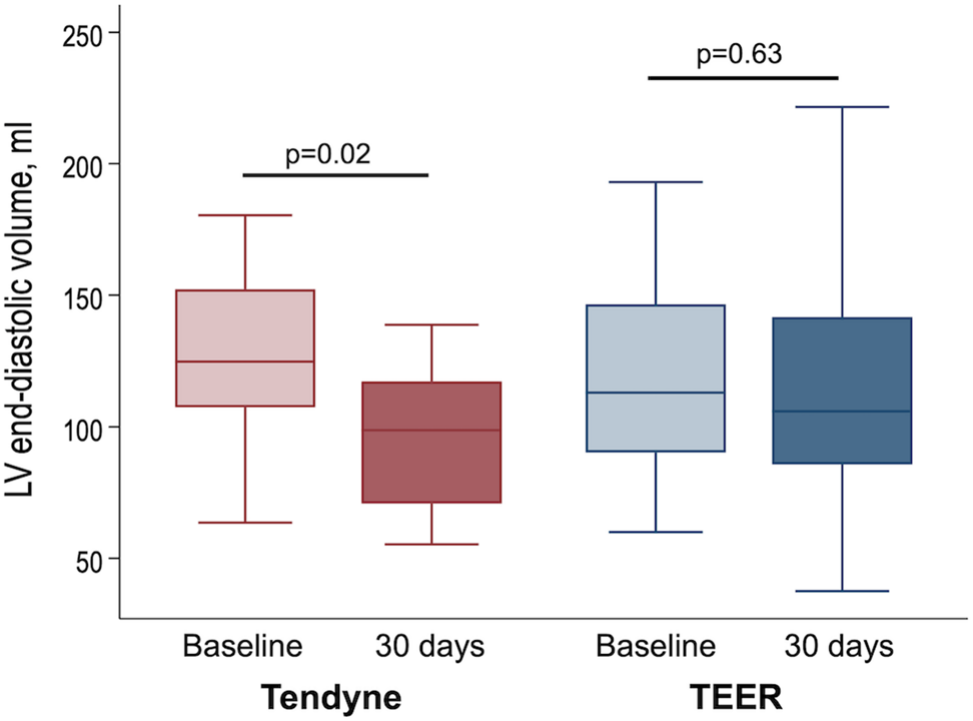
Changes in left ventricular end-diastolic volume after procedure. Changes in left ventricular (LV) end-diastolic volumes from baseline to the one-month follow-up in the Tendyne and TEER groups

Patients treated with Tendyne had a significant reduction in LV end-diastolic volume from baseline to 30 days (124.7 ml [IQR 107.5–152.2] to 98.8 ml [IQR 70.9–117.3]; *p* = 0.022; Fig. 3), and the percent change in LV end-diastolic volume after the Tendyne procedure was 27.3% [IQR  – 0.4 to 41.9]. This reduction in LV end-diastolic volume was greater in the Tendyne group than in the TEER group. In contrast, there were no significant changes in LV end-systolic volume across the groups (Supplemental Fig. 1). Moreover, the reduction in LV longitudinal length was comparable between the Tendyne and TEER groups (3 mm [IQR 2–13] vs. 4 mm [IQR 2–5]; *p* = 0.795).

### Clinical follow-up

Overall, the median follow-up period was 370 days (IQR 255–488). Within one year, six patients treated with the Tendyne (35.3%) died from all causes, including two due to cardiovascular causes. Of these, four patients (23.5%) died within 30 days. We described the detailed information of patients who died within 30 days after the procedure in Supplemental Table 1. Most cases of the 30-day mortality were due to respiratory infection and subsequent sepsis and had comorbidities related to a vulnerability to respiratory infection, such as severe pulmonary disease or end-stage renal dysfunction requiring hemodialysis. Among patients treated with TEER, four patients (8.7%) died within one year, including three due to cardiovascular causes.

**Fig. 4:**
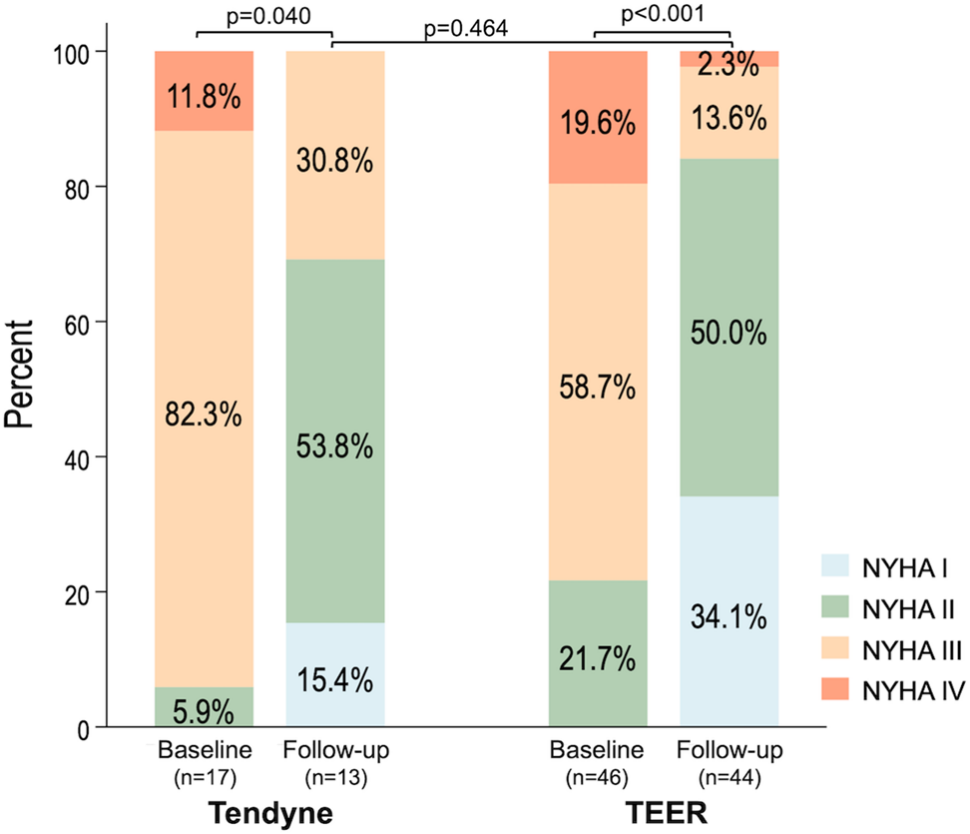
Changes in the New York Heart Association functional class after transcatheter mitral valve intervention. Stacked diagram of the New York Heart Association (NYHA) functional class at baseline and the last follow-up. *TEER*  transcatheter edge-to-edge repair

Data for the New York Heart Association (NYHA) functional class after discharge was available in 13 of 17 patients treated with the Tendyne. The NYHA functional class improved at the final follow-up compared to baseline (Fig. 4), and an improvement by at least one NYHA class was observed in 10 of 13 patients (76.9%) treated with the Tendyne system.

### Inverse probability of treatment weighting analysis

**Fig. 5:**
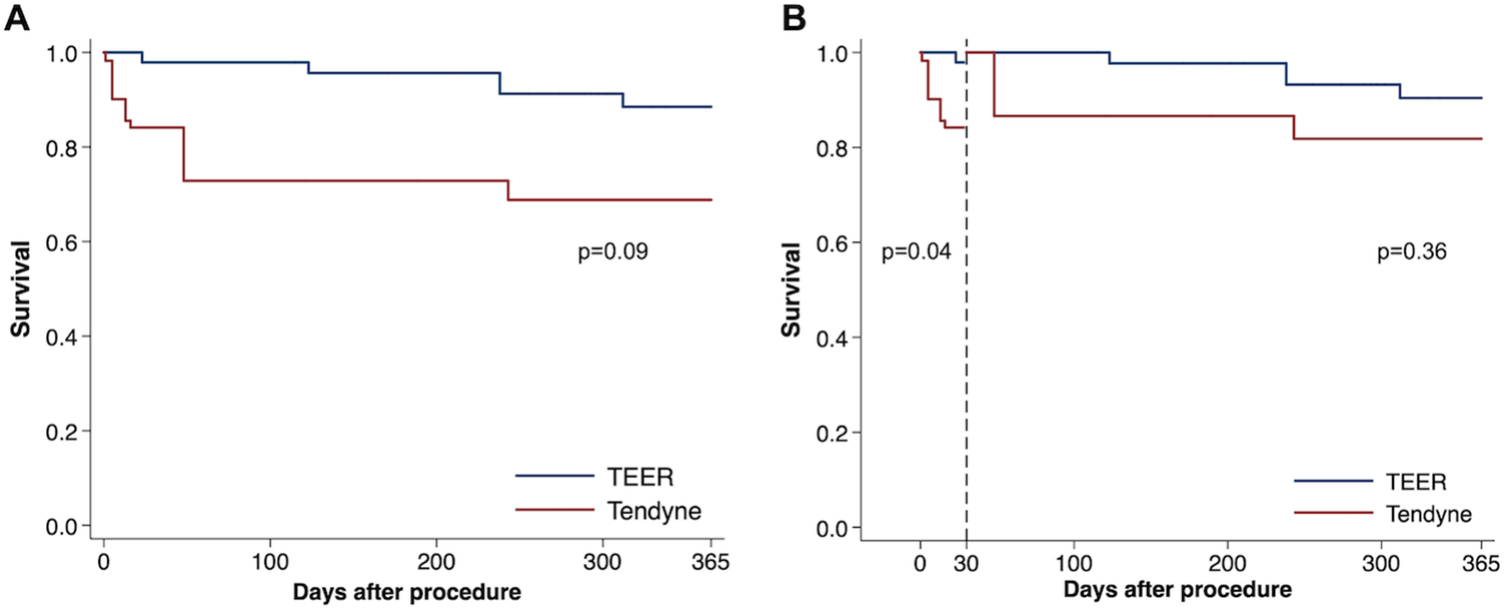
Inverse probability of treatment weighting-adjusted Kaplan–Meier curves of all-cause mortality. Inverse probability of treatment weighting-adjusted Kaplan–Meier curves of all-cause mortality within one year (**A**), up to 30 days, and between 30 days to one year (**B**) in the Tendyne and transcatheter edge-to-edge repair (TEER) groups

We conducted an IPTW analysis to compare clinical outcome between the Tendyne and TEER groups. The propensity scores were differently distributed between patients treated with Tendyne and TEER (Supplemental Fig. 2). After the IPTW adjustment, baseline characteristics were well balanced, with absolute standardized differences less than 20% in most variables (Supplemental Table 2). IPTW-adjusted Kaplan–Meier curves showed that patients treated with the Tendyne system appeared to have a higher all-cause mortality within one year after the procedure, compared to those treated with TEER (*p* = 0.09; Fig. 5). This difference was mostly driven by 30-day mortality (*p* = 0.04), while the mortality from 30 days to one year was comparable between patients treated with the Tendyne system and TEER (*p* = 0.31). Unadjusted Kaplan–Meier curves are shown in Supplemental Fig. 3. The results in the sensitivity analysis were consistent with the main analysis (Supplemental Table 3, Supplemental Figs. 4 and 5).

## Discussion

In this study cohort comprising patients who were considered as feasible for the Tendyne system, we aimed to assess the characteristics and procedural outcomes of TMVR with the Tendyne system in comparison with TEER. The main findings of this study can be summarized as follows:

Of 67 feasible patients for the Tendyne system, 17 underwent TMVR with the Tendyne system, while 46 were treated with TEER. Patients treated with the Tendyne system were likely to be younger and to have a high transmitral pressure gradient and unsuitable mitral valve morphology for TEER, compared with those treated with TEER.

Patients treated with the Tendyne system had a greater reduction in MR and LV end-diastolic volume at the 30-day follow-up than those treated with TEER.

The 30-day mortality after TMVR with the Tendyne system was 23.5%, and most cases of the 30-day mortality were due to non-cardiovascular cause, such as infection.

The IPTW analysis showed that patients treated with the Tendyne system had a higher 30-day mortality compared to those treated with TEER, while the mortality from 30 days to one year was comparable between groups.

TEER is the most widely used transcatheter technique for patients with MR, and the effectiveness of TEER in reducing MR and improving clinical prognosis has been proven. However, it has become increasingly apparent that there are anatomical subsets of the mitral valve that are associated with implantation failure or significant residual MR after TEER [6]. For patients with these anatomical subsets, TEER may be insufficient to achieve an adequate clinical outcome. In contrast, TMVR with the Tendyne system can potentially achieve complete elimination of MR, irrespective of the underlying anatomy and etiology of MR [10, 11], and therefore, TMVR with the Tendyne system could be used to overcome the limitations of TEER in some patients. For a further development of the transcatheter treatment, data on the device selection of transcatheter treatments for MR are needed.

In the present study, we evaluated patients who were considered as eligible for the Tendyne system. For the Tendyne procedure, preprocedural CT is mandatory to assess the anatomy of the mitral valve and evaluate the eligibility of patients for the procedure. Candidates who passed the CT screening for the Tendyne system were included in the present analysis. The study participants were elderly, at a high or prohibitive risk for surgery, and had a high prevalence of significant comorbidities, which was in line with previous reports [7, 10, 11]. In this study population, TMVR with the Tendyne system was performed in 25% of the patients, while approximately 70% of patients were treated with TEER despite the eligibility for the Tendyne system. Patients treated with the Tendyne system were younger and had a higher prevalence of end-stage renal dysfunction requiring hemodialysis than those treated with TEER. In addition, patients treated with the Tendyne system were more likely to have a high transmitral pressure gradient and unsuitable mitral valve anatomy for TEER, which may be associated with a high risk of procedural failure of TEER [6]. Indeed, eight out of 17 patients treated with the Tendyne had a prior history of failed TEER procedures. In the current clinical practice, TMVR with the Tendyne system may be preferred in patients with these clinical and echocardiographic characteristics, and the majority of candidates for the Tendyne system are still treated with TEER.

Despite these anatomical characteristics of the mitral valve, TMVR with the Tendyne system achieved a high technical success rate and reduced the severity of MR to less than 1 + in most patients. This reduction of MR was consistent at the 30-day follow-up, which was greater than in patients treated with TEER. Furthermore, patients treated with the Tendyne had a greater reduction in LV end-diastolic dysfunction at the 30-day follow-up than those treated with TEER. The greater reduction of LV end-diastolic volume may be attributable to a complete correction of MR. Alternatively, the apical tether and epicardial pad at the apex to anchor the Tendyne system may contribute to LV reverse remodeling after the procedure. Fukui et al*.* reported that the location of the epicardial pad affected the reduction of LV end-diastolic volume [20], suggesting that the anchoring system itself may be associated with LV reverse remodeling. This anchoring system might potentially induce an artificial shortening of LV longitudinal length, which could lead to the post-procedural reduction of LV volume. Given the reduction of LV volumes after the MR correction is associated with improved prognosis [21, 22], the durable reduction in MR and the LV reverse remodeling might be an important advantage of the Tendyne system.

Nevertheless, there remains still challenges for TMVR with the Tendyne system. In line with previous studies, TMVR with the Tendyne system had a relatively high 30-day mortality. In the present study, most cases of the 30-day mortality after the Tendyne procedure were due to non-cardiovascular causes (e.g., respiratory infection). These cases of non-cardiovascular mortality had any clinical factors associated with vulnerability to respiratory infection, such as severe pulmonary disease or end-stage chronic kidney disease requiring hemodialysis. Infection was known as one of the major causes of mortality in the early phase after the Tendyne procedure [11]. Vulnerable patients to the acute procedural stress might not be preferable for TMVR with the Tendyne system. In concert with the earlier studies, our current analysis may call for appropriate patient selection to improve the periprocedural safety of the Tendyne system.

The IPTW analysis, which allows to balance the differences in baseline characteristics related to the confounders affecting treatment allocation, showed that the 30-day mortality was higher in the Tendyne group compared with the TEER group, resulting in a higher 1-year mortality in the Tendyne group. In contrast, there was no difference in mortality from 30 days to one year between the groups. Although the greater reduction of MR and LV end-diastolic volume after TMVR with the Tendyne system might have beneficial effects on long-term prognosis, these benefits of TMVR may be counterbalanced by the higher early post-procedural mortality rate. Nonetheless, the current analysis included data of the early experience with the Tendyne system, which might be associated with a high rate of adverse events. Therefore, future prospective randomized trials are warranted to compare outcomes between TMVR with the Tendyne system and TEER, such as the SUMMIT trial (NCT03433274). Furthermore, it is also necessary to elucidate the factors associated with peri-procedural adverse events of TMVR with the Tendyne system and to refine patient selection and peri-procedural management strategies based on these factors.

### Limitations

First, given that this was a retrospective analysis using data from a single-center and relatively small cohort, there may be several potential selection biases of the study population and treatment allocation. Since this study included patients who underwent the CT screening for the Tendyne system, approximately two thirds of the study population had conditionally suitable or unsuitable valve morphology for TEER. Moreover, because of the retrospective fashion, there was no predefined criteria for the device selection, and the treatment allocation was decided by the heart team based on multiple factors, such as clinical and echocardiographic characteristics. Secondly, we attempted to correct for the bias in treatment allocation by using an IPTW-adjusted approach to compare clinical outcomes between TMVR with the Tendyne system and TEER; however, our findings should be interpreted with caution for four major reasons: (1) the adjustment with IPTW approach might be insufficient because of the limited number of patients; (2) there might be unmeasured factors related to the device selection, including the morphology of mitral valve; and (3) eight of the 17 patients in the Tendyne group had a prior history of an unsuccessful TEER procedure, which could not be adjusted in the present analysis. Nevertheless, this is the first study investigating the difference in clinical prognosis and echocardiographic findings between Tendyne and TEER in patients eligible for the Tendyne system. To validate our finding, future prospective randomized trials are warranted. Finally, the echocardiographic assessments performed at the 30-day follow-up did not include patients who died before the follow-up, which could also affect our results.

## Conclusion

Of potential candidates for the Tendyne system, 25% of patients underwent TMVR with the Tendyne system, while the majority of the patients were still treated with TEER. Despite a high transmitral pressure gradient at baseline and unsuitable mitral valve morphology for TEER, TMVR with the Tendyne system showed a substantial reduction of MR, with residual MR < 1 + in 94.1% of patients. Furthermore, LV reverse remodeling during the follow-up was more pronounced after TMVR compared to TEER. However, patients treated with TMVR the Tendyne system still appeared to have a higher all-cause mortality within one year compared to TEER, which was mainly driven by the 30-day mortality rate. Appropriate patient selection and further development of the TMVR technology may be necessary to improve periprocedural safety and clinical outcomes.

### Supplementary Information

Below is the link to the electronic supplementary material.

Supplementary file1 (DOCX 3899 KB)

## Data Availability

All the data underlying this article are available in the article and in its online supplementary material.

## Abbreviations

IQR: Interquartile range
IPTW: Inverse probability of treatment weighting
LVEF: Left-ventricular ejection fraction
LV: Left ventricle
MR: Mitral regurgitation
NYHA: New York Heart Association
TEER: Transcatheter edge-to-edge repair
TMVR: Transcatheter mitral valve replacement
TEE: Transesophageal echocardiography
TTE: Transthoracic echocardiography
