# Correction: Characteristics and outcomes of patients undergoing transcatheter mitral valve replacement with the Tendyne system

**DOI:** 10.1007/s00392-023-02174-8

**Published:** 2023-02-27

**Authors:** Nihal Wilde, Tetsu Tanaka, Vivian Vij, Atsushi Sugiura, Mitsumasa Sudo, Eva Eicheler, Miriam Silaschi, Johanna Vogelhuber, Farhad Bakhtiary, Georg Nickenig, Marcel Weber, Sebastian Zimmer

**Affiliations:** 1 Heart Center Bonn, Department of Internal Medicine II, University Hospital Bonn, Venusberg-Campus 1, 53127 Bonn, Germany; 2 Heart Center Bonn, Department of Cardiac Surgery, University Hospital Bonn, Bonn, Germany

**Correction: Clinical Research in Cardiology** 10.1007/s00392-023-02155-x

In the original article, the author name Tetsu Tanaka was given incorrectly as ‘Tetsu Tenaka’. The original article has been corrected.

