# Supplementary material for: Characteristics and outcomes of patients undergoing transcatheter mitral valve replacement with the Tendyne system
Source: Clin Res Cardiol. 2023 Jan 16;113(1):1–10. doi: 10.1007/s00392-023-02155-x (PMC10808407; doi:10.1007/s00392-023-02155-x)
Supplement: Supplementary file 1 — Supplementary file1 (DOCX 3899 KB) [file 392_2023_2155_MOESM1_ESM.docx]

**Supplemental materials**

**Supplemental Methods**

*Inverse probability of treatment weighting analysis*

Each patient was weighted by the inverse probability of being in the observed group: patients who underwent TMVR with the Tendyne system were weighted by the reciprocal of the propensity score, and those who underwent TEER were weighted by the reciprocal of 1 minus the propensity score; the weights were then stabilized by the proportion of patients in each group. After IPTW, survival analyses were conducted using Kaplan-Meier curves. Furthermore, we applied IPTW to estimate the average treatment effects of TMVR with the Tendyne system on the percent change in LV volumes at the 30-day follow-up.

As a sensitivity analysis, we calculated the propensity score using another multivariable logistic regression model including the following covariates: the covariates in the main model, posterior leaflet length <10 mm, tenting height ≥11 mm, and moderate or severe mitral annular calcification. Clinical outcomes and average treatment effects of TMVR on the percent changes in LV volumes were similarly compared using this propensity score, as the main analysis.

**Supplemental Figure 1. Changes in left-ventricular end-systolic volume at 30 days after the procedure**


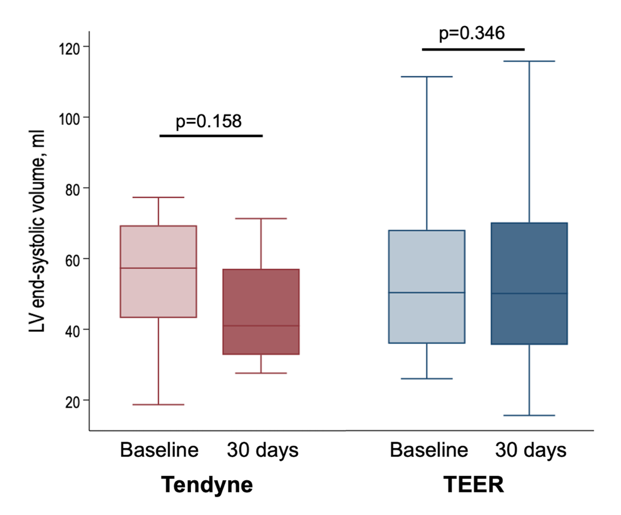


Legend: Changes in left-ventricular (LV) end-systolic volume from baseline to the one-month follow-up in the Tendyne and transcatheter edge-to-edge repair (TEER) groups.

**Supplemental Figure 2. Distribution of propensity score**


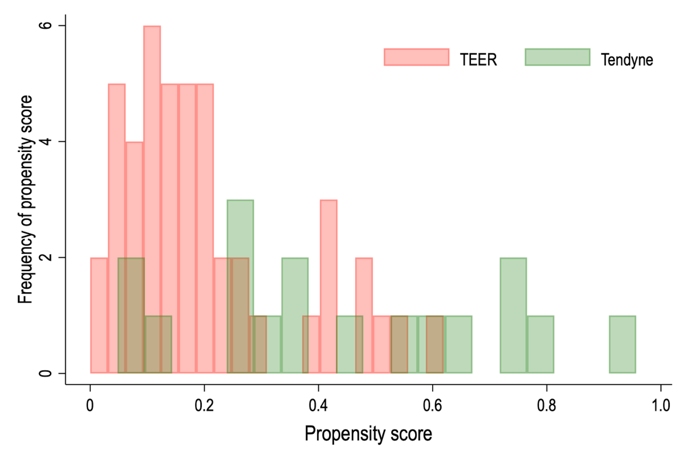


Legend: Distribution of the propensity score in the Tendyne and transcatheter edge-to-edge repair (TEER) groups.

**Supplemental Figure 3. Unadjusted Kaplan–Meier curves of all-cause mortality after the procedures**


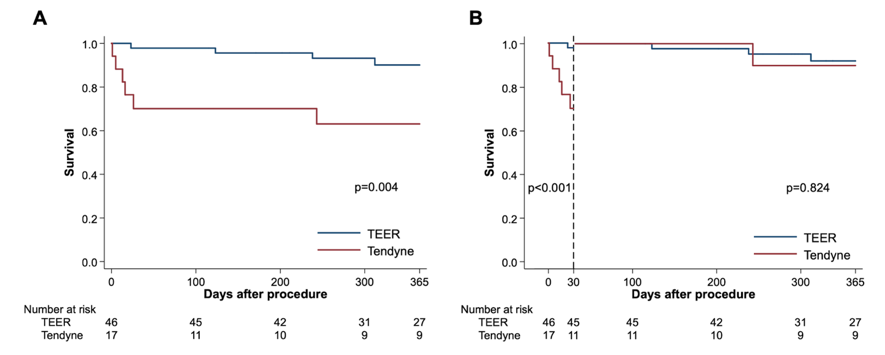


Legend: Unadjusted Kaplan–Meier curves of all-cause mortality within one year (A), within 30 days, and between 30 days to one year (B) in the Tendyne and transcatheter edge-to-edge repair (TEER) groups.

**Supplemental Figure 4. Distribution of propensity scores in sensitivity analysis**


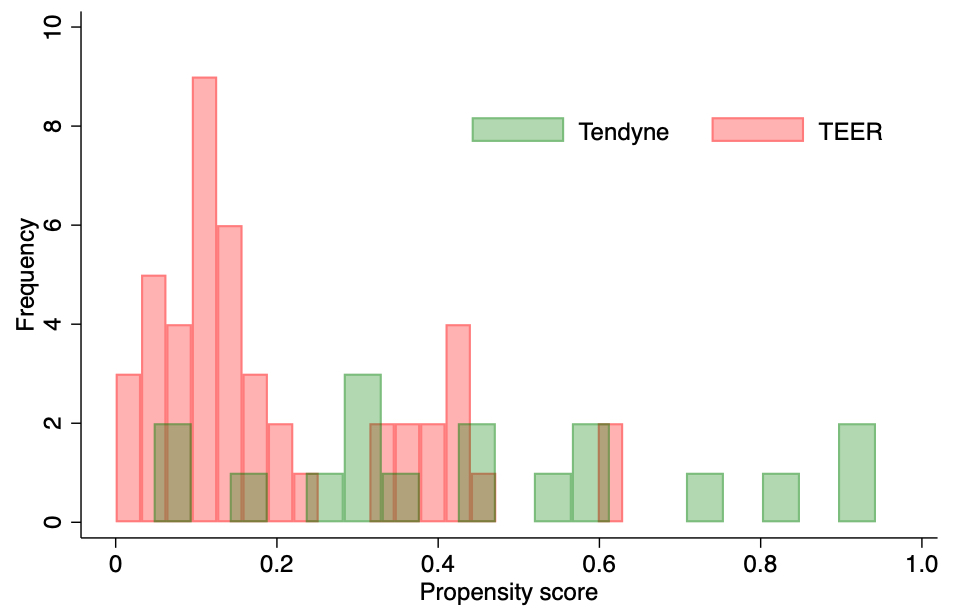


Legend: Distribution of the propensity score in the Tendyne and transcatheter edge-to-edge repair (TEER) groups in the sensitivity analysis.

**Supplemental Figure 5. IPTW-adjusted Kaplan–Meier curves for all-cause mortality in sensitivity analysis**

**
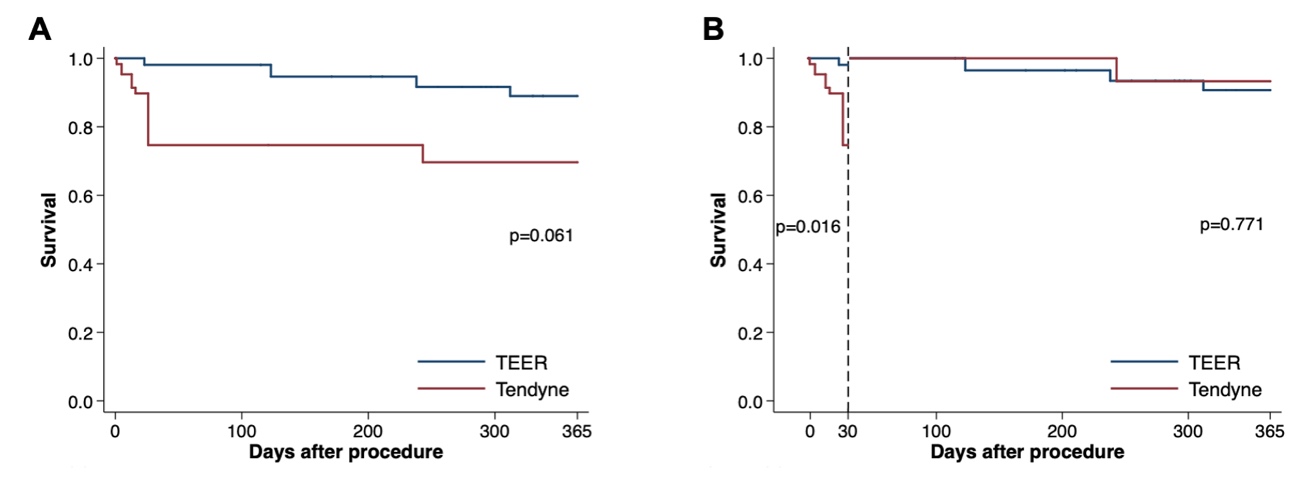
**

Legend: IPTW-adjusted Kaplan–Meier curves of all-cause mortality within one year (A), within 30 days, and between 30 days to one year (B) in the Tendyne and transcatheter edge-to-edge repair (TEER) groups in the sensitivity analysis.

**Supplemental Table 1. Detailed information of 30-day mortality after the Tendyne procedure**

| Case No. | Age, years | Sex | Comorbidities | Days of mortality from procedure | Cause of death |
| --- | --- | --- | --- | --- | --- |
| 1 | 72 | Female | End-stage renal dysfunction requiring hemodialysis | 5 days | Sepsis due to pulmonary infection |
| 2 | 49 | Female | A prior history of acute respiratory dysfunction syndrome requiring extracorporeal circulation | 16 days | Sepsis due to pulmonary infection |
| 3 | 86 | Female | Insulin-dependent diabetes mellitus | 28 days | Cerebral infarction and sepsis due to urinary infarction |
| 4 | 64 | Female | End-stage renal dysfunction requiring hemodialysis | 13 days | Sepsis due to pulmonary infection |
| 5 | 73 | Male | Very low left-ventricular ejection fraction (20%) | 1 day | Multi-organ dysfunction after a conversion to surgery due to cardiac tamponade |

**Supplemental Table 2. Baseline characteristics before and after inverse probability of treatment weighting**

|  | Before IPTW | | | After IPTW | | |
| --- | --- | --- | --- | --- | --- | --- |
|  | Tendyne | TEER | Absolute SD | Tendyne | TEER | Absolute SD |
|  | N=17 | N=46 |  |  |  |  |
| Age, years | 72.9 ± 9.4 | 78.1 ± 6.9 | 61.9 | 77.7 ± 7.2 | 77.6 ± 6.9 | 2.0 |
| Male, % | 47.1 | 45.7 | 2.8 | 45.8 | 47.2 | 2.8 |
| BMI, kg/m^2^ | 30.7 ± 7.4 | 26.1 ± 5.6 | 67.3 | 26.4 ± 6.1 | 26.8 ± 5.9 | 6.3 |
| CAD, % | 52.9 | 58.7 | 11.4 | 53.0 | 60.1 | 14.0 |
| Prior MI, % | 11.8 | 13.0 | 3.8 | 27.0 | 12.7 | 42.5 |
| Prior cardiac surgery, % | 29.4 | 28.3 | 21.1 | 18.0 | 26.3 | 18.0 |
| COPD, % | 29.4 | 32.6 | 16.5 | 12.1 | 34.5 | 50.7 |
| NYHA IV, % | 11.8 | 19.6 | 21.2 | 8.6 | 17.3 | 25.9 |
| Atrial fibrillation, % | 76.5 | 78.3 | 4.2 | 56.7 | 75.6 | 44.1 |
| CIED, % | 23.5 | 23.9 | 0.9 | 33.7 | 22.1 | 25.0 |
| eGFR, ml/min/m^2^ | 53.0 [34.8, 66.8] | 53.9 [40.8, 72.6] | 26.1 | 62.0 [34.8, 75.6] | 53.9 [40.0, 77.2] | 12.0 |
| NT-proBNP, pg/ml | 1582 [1285, 5035] | 2528 [1175, 3953] | 37.8 | 1343 [364, 1835] | 2770 [1175, 3744] | 17.6 |
| EuroSCORE II, % | 3.37 [2.07, 6.35] | 3.74 [2.16, 5.57] | 13.3 | 3.49 [2.05, 6.68] | 3.67 [2.15, 5.23] | 0.2 |
| Beta blockers, % | 70.6 | 89.1 | 46.4 | 66.7 | 87.6 | 52.2 |
| RAS inhibitors, % | 82.4 | 80.4 | 4.8 | 65.1 | 78.8 | 34.5 |
| Echocardiographic findings |  |  |  |  |  |  |
| LVEF, % | 55.5 [52.2, 58.0] | 55.7 [43.1, 59.5] | 20.2 | 55.5 [42.1, 65.3] | 56.0 [45.7, 60.0] | 19.3 |
| LV end-diastolic volume, ml | 124.7 [107.5, 152.2] | 114.3 [91.0, 146.5] | 23.5 | 124.7 [96.8, 135.0] | 114.3 [91.0, 146.5] | 1.7 |
| LV end-systolic volume, ml | 57.3 [43.2, 69.4] | 60.4 [37.0, 68.1] | 2.1 | 52.2 [39.9, 71.3] | 49.0 [35.9, 66.5] | 14.9 |
| LV length, mm | 81 [77, 84] | 77 [72, 83] | 18.5 | 81 [77, 84] | 78 [72, 84] | 41.4 |
| LA volume, ml | 100.0 [77.9, 120.0] | 90.0 [61.2, 117.8] | 43.2 | 120 [86.6, 135.0] | 89.9 [65.0, 113.1] | 61.2 |
| Etiology of MR: FMR, % | 47.1 | 69.6 | 37.0 | 55.8 | 57.4 | 3.2 |
| MR severity, % |  |  | 4.2 |  |  | 14.6 |
| 3+ | 35.3 | 39.1 |  | 49.1 | 41.2 |  |
| 4+ | 64.7 | 60.9 |  | 50.9 | 58.8 |  |
| EROA, mm^2^ | 38 [31, 42] | 34 [29, 41] | 24.0 | 40 [33, 40] | 34 [29, 42] | 41.0 |
| Regurgitant volume, ml | 56 [41, 76] | 55 [45, 71] | 25.7 | 42 [42, 56] | 56 [45, 74] | 67.4 |
| Mean transmitral pressure gradient, mmHg | 2.2 [1.8, 4.0] | 1.8 [1.1, 2.7] | 37.5 | 1.5 [1.4, 2.2] | 1.9 [1.1, 2.7] | 11.3 |
| Short posterior leaflet length <10mm | 35.3 | 19.6 | 63.0 | 39.5 | 18.8 | 122.0 |
| Coaptation depth ≥11mm | 5.9 | 10.9 | 2.6 | 2.6 | 10.4 | 0.05 |
| SPAP, mmHg | 40 [38, 45] | 41 [28, 47] | 19.7 | 40 [29, 45] | 42 [31, 47] | 32.9 |
| TAPSE, mm | 20 [15, 27] | 20 [15, 22] | 16.8 | 24 [16, 29] | 20 [16, 22] | 48.3 |
| TR: severe or more, % | 29.4 | 37.0 | 9.5 | 26.0 | 34.2 | 8.0 |
| Values are either %, mean ± SD, or median [interquartile range].  Legends: IPTW = inverse probability of treatment weighting; SD = standardized difference. | | | | | | |

**Supplemental Table 3. Baseline characteristics before and after inverse probability of treatment weighting in the sensitivity analysis**

|  | Before IPTW | | | After IPTW | | |
| --- | --- | --- | --- | --- | --- | --- |
|  | Tendyne | TEER | Absolute SD | Tendyne | TEER | Absolute SD |
|  | N=17 | N=46 |  |  |  |  |
| Age, years | 72.9 ± 9.4 | 78.1 ± 6.9 | 61.9 | 78.2 ± 7.5 | 77.7± 6.9 | 5.9 |
| Male, % | 47.1 | 45.7 | 2.8 | 33.0 | 47.2 | 27.9 |
| BMI, kg/m^2^ | 30.7 ± 7.4 | 26.1 ± 5.6 | 67.3 | 26.2 ± 6.2 | 26.6 ± 5.7 | 6.3 |
| CAD, % | 52.9 | 58.7 | 11.4 | 38.8 | 61.1 | 44.1 |
| Prior MI, % | 11.8 | 13.0 | 3.8 | 12.3 | 12.9 | 1.8 |
| Prior cardiac surgery, % | 29.4 | 28.3 | 21.1 | 18.4 | 26.3 | 16.9 |
| COPD, % | 29.4 | 32.6 | 16.5 | 12.7 | 33.0 | 43.0 |
| NYHA IV, % | 11.8 | 19.6 | 21.2 | 8.1 | 17.2 | 24.8 |
| Atrial fibrillation, % | 76.5 | 78.3 | 4.2 | 62.7 | 77.0 | 33.5 |
| CIED, % | 23.5 | 23.9 | 0.9 | 21.3 | 23.9 | 5.8 |
| eGFR, ml/min/m^2^ | 53.0 [34.8, 66.8] | 53.9 [40.8, 72.6] | 26.1 | 53.0 [34.8, 66.8] | 53.7 [40.0, 77.2] | 23.9 |
| NT-proBNP, pg/ml | 1582 [1285, 5035] | 2528 [1175, 3953] | 37.8 | 1343 [1297, 1835] | 2770 [1175, 3744] | 7.1 |
| EuroSCORE II, % | 3.37 [2.07, 6.35] | 3.74 [2.16, 5.57] | 13.3 | 6.68 [2.71, 11.20] | 3.67 [2.17, 5.23] | 17.2 |
| Beta blockers, % | 70.6 | 89.1 | 46.4 | 62.2 | 88.6 | 64.2 |
| RAS inhibitors, % | 82.4 | 80.4 | 4.8 | 52.6 | 78.9 | 66.3 |
| Echocardiographic findings |  |  |  |  |  |  |
| LVEF, % | 55.5 [52.2, 58.0] | 55.7 [43.1, 59.5] | 20.2 | 58.0 [52.2, 65.3] | 56.5 [45.7, 60.0] | 51.7 |
| LV end-diastolic volume, ml | 124.7 [107.5, 152.2] | 114.3 [91.0, 146.5] | 23.5 | 124.7 [86.5, 133.4] | 114.3 [91.0, 146.5] | 14.1 |
| LV end-systolic volume, ml | 57.3 [43.2, 69.4] | 60.4 [37.0, 68.1] | 2.1 | 43.2 [36.3, 67.7] | 50.3 [35.9, 66.5] | 39.1 |
| LV length, mm | 81 [77, 84] | 77 [72, 83] | 18.5 | 77 [73, 83] | 78 [72, 84] | 21.3 |
| LA volume, ml | 100.0 [77.9, 120.0] | 90.0 [61.2, 117.8] | 43.2 | 120.0 [98.2, 135.0] | 89.9 [65.0, 113.1] | 66.5 |
| Etiology of MR: FMR, % | 47.1 | 69.6 | 37.0 | 46.9 | 56.3 | 19.1 |
| MR severity, % |  |  | 4.2 |  |  | 7.8 |
| 3+ | 35.3 | 39.1 |  | 43.4 | 39.6 |  |
| 4+ | 64.7 | 60.9 |  | 56.6 | 60.4 |  |
| EROA, mm^2^ | 38 [31, 42] | 34 [29, 41] | 24.0 | 38 [31, 40] | 35 [29, 45] | 40.7 |
| Regurgitant volume, ml | 56 [41, 76] | 55 [45, 71] | 25.7 | 42 [41, 58] | 56 [45, 76] | 55.9 |
| Mean transmitral pressure gradient, mmHg | 2.2 [1.8, 4.0] | 1.8 [1.1, 2.7] | 37.5 | 1.5 [1.0, 2.2] | 1.9 [1.1, 2.8] | 13.4 |
| Short posterior leaflet length <10mm | 35.3 | 19.6 | 63.0 | 22.9 | 22.9 | 0.1 |
| Coaptation depth ≥11mm | 5.9 | 10.9 | 2.6 | 3.4 | 9.7 | 22.5 |
| SPAP, mmHg | 40 [38, 45] | 41 [28, 47] | 19.7 | 40 [25, 44] | 42 [31, 47] | 18.8 |
| TAPSE, mm | 20 [15, 27] | 20 [15, 22] | 16.8 | 20 [16, 30] | 20 [16, 22] | 50.2 |
| TR: severe or more, % | 29.4 | 37.0 | 9.5 | 34.0 | 33.7 | 0.6 |
| Values are either %, mean ± SD, or median [interquartile range].  Legends: IPTW = inverse probability of treatment weighting; SD = standardized difference; BMI = body mass index; CAD = coronary artery disease; MI = myocardial infarction; COPD = chronic obstructive pulmonary disease; NYHA = New York Heart Association; CIED = cardiac implantable electronic device; eGFR = estimated glomerular filtration rate; NT-proBNP = N-terminal pro-B-type natriuretic peptide; EuroSCORE = European System for Cardiac Operative Risk Evaluation; RAS = renin-angiotensin system; LVEF = left-ventricular ejection fraction; FMR = functional mitral regurgitation; SPAP = systolic pulmonary artery pressure; TAPSE = tricuspid annular plane systolic excursion; TR = tricuspid regurgitation. | | | | | | |
